# Cervical Ectopic Pregnancy in a 23 Year Old with Uterus Didelphys

**DOI:** 10.5811/cpcem.2016.11.33052

**Published:** 2017-01-18

**Authors:** Patrick C. Ng, Kristen S. Kann

**Affiliations:** San Antonio Uniformed Services Health Education Consortium, Department of Emergency Medicine, San Antonio, Texas

## Abstract

Ectopic pregnancy remains an important diagnosis for the emergency physician to recognize, accounting for up to 2% of all pregnancies and associated with significant morbidity and mortality. Ectopic pregnancies can implant in various sites outside of the uterus, one of the rarest of which is in the cervix. Cervical ectopics account for less than 1% of ectopic pregnancies, but are associated with higher rates of significant bleeding than others.^1^–^2^ Uterine anomalies are a predisposing factor for ectopic pregnancies. This case highlights the management of a cervical ectopic pregnancy in a 23 year old with a history of uterine didelphys.

## INTRODUCTION

Ectopic pregnancy is an important diagnosis to consider in a fertile female who presents to the emergency department with vaginal bleeding and/or abdominal pain. It is defined as the implantation of the zygote outside of the uterine cavity. Ectopic pregnancies are associated with various complications that can lead to significant morbidity and mortality if not promptly and appropriately managed^1^. Hemorrhage from ectopic pregnancy remains the leading cause of mortality in the first trimester.

Ectopic pregnancies can occur in multiple places. 98% of ectopic gestations occur in the fallopian tubes. Cervical pregnancy is rare, accounting for <1% of ectopic pregnancies^2^. Due to a paucity of information on cervical ectopic pregnancies, the most effective treatment has not been clearly defined.

Generally, there are two mainstays of treatment of ectopic pregnancies; surgical and medical. Surgical therapy involves manually extracting an ectopic pregnancy and any other affected anatomic structures as clinically indicated. Medical therapy involves the administration of an abortive medication. Currently, the most common agent used is methotrexate, a folic acid antagonist that inhibits DNA synthesis in actively dividing cells^3^. Several studies have demonstrated that methotrexate was as effective as laparoscopic treatment of ectopic pregnancies when used appropriately^4^–^6^. (Table 1)

Risk factors for ectopic pregnancies include anatomic anomalies, prior ectopic pregnancies, in utero diethylstilbestrol exposure, implantation in the presence of an intrauterine device, smoking, previous genital infections, and in vitro fertilization. Uterine anomalies arise from abnormal embryological development of the Mullerian ducts. Of the Mullerian duct abnormalities, a didelphic uterus is one of the rarest, making up just 8.3% of all Mullerian duct abnormalities^7^. A didelphic uterus results from incomplete failure of the Mullerian ducts to fuse resulting in two separate uterine cavities, two cervices, and often vaginal abnormalities such as double vagina or a longitudinal vaginal septum^14^. It can be associated with Wolffian duct abnormalities resulting in kidney abnormalities as well.

We report a case of a 23 year old female who presented with vaginal bleeding and was found to have a cervical ectopic pregnancy and a didelphic uterus.

## CASE REPORT

A 23 year old female G1P0, with a history of hypothyroidism and polycystic kidney disease presented to the ED with 1 week of pelvic pain and 1 day of vaginal bleeding. She described the pelvic pain as crampy and intermittent with no specific relieving or exacerbating features. The day prior to presentation, she noticed heavy vaginal bleeding and described the passage of clots and tissue.

**Image 1:**
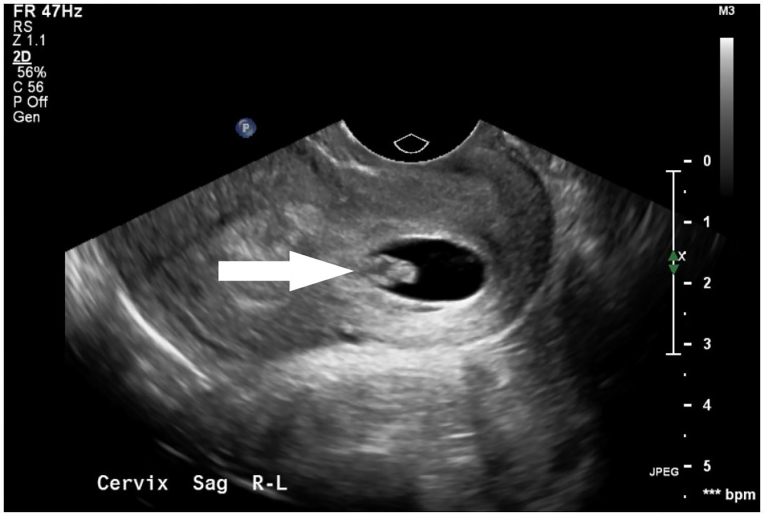
Transvaginal ultrasound demonstrating two uterine cavities (arrows).

**Image 2:**
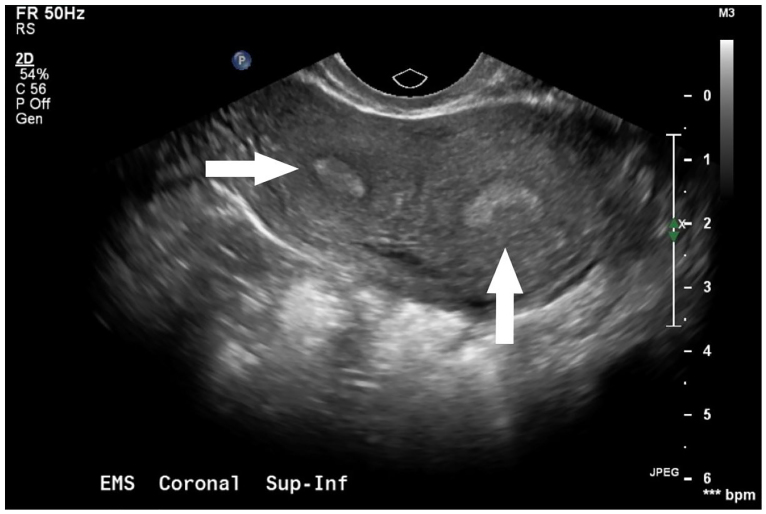
Transvaginal ultrasound demonstrating a gestational sac (arrow) in the left endocervical canal.

Upon presentation, she was hemodynamically normal. Her physical exam was notable for mild lower abdominal tenderness to palpation. Her pelvic exam revealed normal appearing external female genitalia, a normal appearing cervix in the midline, and a smaller cervix to the left, without obvious products of conception. There was evidence of a small partial longitudinal septation between the two cervices. Bimanual exam was unremarkable. Laboratory evaluation was significant for a serum beta hCG of 6,045 mIU/mL, B positive blood type, a hemoglobin of 13.7 g/dL with a hematocrit of 41.5%. Transvaginal ultrasound was notable for two uteri, two cervices and a gestational sac without yolk sac or fetal pole present in the left endocervical canal (Image 1, Image 2).

The patient was diagnosed with a cervical ectopic pregnancy, and OB/GYN was consulted and evaluated the patient at bedside. The patent was discharged home with 48 hour follow up with OB/GYN. Repeat ultrasound in clinic showed persistence of gestational sac in left endocervical canal as noted in prior imaging study. Methotrexate therapy was initiated, and the patient received 81mg IM × 1 in clinic 2 days after she was seen in the Emergency Department. She had weekly follow-up with repeat B-HCG levels (Table 2) following the methotrexate dose. By week 4, her β-hCG levels and repeat ultrasound revealed resolution of the cervical ectopic. The patient was counseled on birth control and to seek early OB care if pregnant again in the future.

## DISCUSSION

To our knowledge, this is the first reported case of a cervical ectopic in a patient with a didelphic uterus in the emergency medicine literature. Ectopic pregnancies account for 6–16% of all causes of first trimester bleeding^3^. Hemorrhage resulting from ectopic pregnancy is the leading cause of maternal death in the first trimester and has been reported to be up to 10% of all pregnancy-related deaths^8^. Most ectopic pregnancies occur in the fallopian tube and <1% occur in the cervix. Cervical ectopics are important to recognize in the Emergency Department due to the increased risk of life-threatening bleeding due to erosion into the cervical blood vessels. Predisposing factors for cervical ectopic pregnancies include prior dilation and curettage, prior caesarean section, and in vitro fertilization. While this patient presented in stable condition, some ectopic pregnancies can present with severe vaginal bleeding. Emergent therapies beyond rapid consultation with a gynecologic surgeon include foley balloon tamponade of the cervix and vaginal packing.

There are many risk factors that predispose individuals to developing ectopic pregnancies. It is important for the emergency physician to be aware of such risk factors, including rare conditions such as a didelphic uterus. Emergency providers should be cognizant of didelphic uterus due to the expected abnormalities on physical exam, as well as the importance to fully visualize all of the relevant anatomy on ultrasound.

The optimal management of cervical ectopic pregnancy in a patient with a didelphic uterus remains unclear. Due the paucity of information present on cervical ectopic pregnancies in patients with a didelphic uterus, it is unknown whether or not medical or surgical management is favored^2^. The information presented on management of cervical ectopic is limited to isolated case reports and small retrospective studies^9^–^12^. Many of these reports discuss the use of medical therapy of cervical ectopic with a secondary goal of preserving the uterus for potential future pregnancies. However, none of these reports discuss the management of cervical ectopic pregnancy in a patient with a history of didelphic uterus and the fertility of individuals who have this anatomy is not well defined^13^. This case demonstrates the successful management of a rare presentation of ectopic pregnancies with single dose methotrexate. In a hemodynamically normal patient with close follow-up and mild to moderate bleeding, it may be appropriate to discharge these patients from the ED with strict return precautions. As with most ectopic pregnancies, we recommend expert consultation with OB/GYN.

## Figures and Tables

**Table 1:** Favorable criteria for medical management of ectopic pregnancy.

Criteria
Stable hemodynamics
Compliance
Post-treatment follow-up
Human chorionic gonadotropin concentration ≤5000mlU/mL
No fetal cardiac activity
Ectopic mass size ≤3cm

**Table 2:** Human chorionic gonadotropin concentration (hCG) of the patient from November 7 to December 7.

Date(2015)	hCG concentration mIU/mL
November 7	6045
November 9	2789
November 13	675.7
November 16	265.9
November 23	52.9
November 30	8.9
December 7	3.1
